# Crystal structure of the eukaryotic translation initiation factor 2A from *Schizosaccharomyces pombe*

**DOI:** 10.1007/s10969-014-9177-y

**Published:** 2014-02-26

**Authors:** Kazuhiro Kashiwagi, Takuhiro Ito, Shigeyuki Yokoyama

**Affiliations:** 1 Department of Biophysics and Biochemistry, Graduate School of Science, The University of Tokyo, 7-3-1 Hongo, Bunkyo-ku, Tokyo, 113-0033 Japan; 2 RIKEN Systems and Structural Biology Center, 1-7-22 Suehiro-cho, Tsurumi-ku, Yokohama-shi, Kanagawa 230-0045 Japan; 3 RIKEN Structural Biology Laboratory, 1-7-22 Suehiro-cho, Tsurumi-ku, Yokohama-shi, Kanagawa 230-0045 Japan; 4 RIKEN Center for Life Science Technologies, 1-7-22 Suehiro-cho, Tsurumi-ku, Yokohama-shi, Kanagawa 230-0045 Japan

**Keywords:** Translation initiation factor, eIF2A, β-propeller protein, X-ray crystal structure

## Abstract

The eukaryotic translation initiation factor 2A (eIF2A) was identified as a factor that stimulates the binding of methionylated initiator tRNA (Met-tRNA_i_^Met^) to the 40S ribosomal subunit, but its physiological role remains poorly defined. Recently, eIF2A was shown to be involved in unconventional translation initiation from CUG codons and in viral protein synthesis under stress conditions where eIF2 is inactivated. We determined the crystal structure of the WD-repeat domain of *Schizosaccharomyces pombe* eIF2A at 2.5 Å resolution. The structure adopts a novel nine-bladed β-propeller fold. In contrast to the usual β-propeller proteins, the central channel of the molecule has the narrower opening on the bottom of the protein and the wider opening on the top. Highly conserved residues are concentrated in the positively-charged top face, suggesting the importance of this face for interactions with nucleic acids or other initiation factors.

## Introduction

In the 1970s, translation-initiation-complex formation in eukaryotes was found to be divided into two pathways, which differ with respect to the loading of the methionylated initiator tRNA (Met-tRNA_i_^Met^) onto the 40S ribosomal subunit [1]. In one pathway, which is mediated by the eukaryotic translation initiation factor (eIF) 2, Met-tRNA_i_^Met^ is loaded onto the ribosome in a guanosine-triphosphate (GTP)-dependent and mRNA-independent manner. In the other pathway, which is mediated by eIF2A, the loading requires the trinucleotide AUG start codon, but not GTP. Due to its AUG-dependence, eIF2A was initially considered to be a eukaryotic functional counterpart of the prokaryotic translation initiation factor IF2 [2]. However, methionyl-puromycin synthesis assays revealed that the eIF2-mediated pathway is far more efficient, and only eIF2 is able to form the 80S initiation complex on native globin mRNA [1]. Furthermore, yeast strains lacking eIF2A are viable, and their only apparent phenotype is reduced sporulation [3]. These findings suggested that eIF2 is responsible for the loading of Met-tRNA_i_^Met^ onto the ribosome in the majority of translation initiation events in eukaryotes, while eIF2A is involved in the translation of a limited subset of mRNAs.

Homologues of eIF2A are found in a wide range of eukaryotic species, from yeast to human, indicating that eIF2A plays some conserved roles. Recently, eIF2A was reported to be involved in antigen presentation by major histocompatibility complex (MHC) class I molecules [4]. Portions of the antigenic precursors are translated from CUG codons via an unknown mechanism, which is different from the conventional methionine-initiated mechanism [5]. The presentation of CUG/leucine-initiated peptides was inhibited by the knockdown of eIF2A, but insensitive to reduced eIF2-Met-tRNA_i_^Met^ levels. Ribosome profiling experiments also revealed that translation initiation at CUG or GUG codons is a common event in yeast and human [6, 7], and is not limited to antigen presentation by MHC class I molecules. Reduced eIF2-Met-tRNA_i_^Met^ levels are caused by various stresses, including amino acid starvation, heat shock and viral infection. Under such conditions, eIF2A migrates from the nucleus to the cytoplasm [8], where it is utilized for the translation of alphavirus 26S mRNA [9] and hepatitis C viral (HCV) mRNA [8]. As for HCV, the IIId domain of the HCV internal ribosome entry site (IRES) binds eIF2A, and the infectivity is reduced by the knockdown of eIF2A [8]. Based on these findings, eIF2A might be a tRNA-loading factor in unconventional translation initiation events, such as non-AUG translation initiation, and an alternative factor of eIF2 when the active eIF2 is limited, due to the stress response by eIF2 kinases.

eIF2A is predicted to contain two to four WD-repeats at the N-terminus and a coiled-coil at the C-terminus, but this prediction has not been structurally confirmed. Here we present the crystal structure of the C-terminally truncated eIF2A, from the fission yeast *Schizosaccharomyces pombe*. This truncated eIF2A forms a β-propeller fold, containing nine blades. This is the first report of the structure of eIF2A and a nine-bladed β-propeller protein.

## Materials and methods

### Expression and purification of *S. pombe* eIF2A

The region encoding the *S. pombe* eIF2A gene was cloned between the *Nhe*I and *Not*I sites of the pET-28c vector (Novagen). For the C-terminally truncated eIF2A, a TAG codon was inserted at the desired position with a PrimeSTAR Mutagenesis Basal Kit (Takara). *E. coli* Rosetta2(DE3) cells were transformed with the plasmid, and grown at 37 °C in M9 medium. When the OD_600_ reached 0.6, the medium was supplemented with selenomethionine (SeMet) and amino acids [10], and the temperature was lowered to 18 °C. After 30 min, protein expression was induced with 0.5 mM isopropyl-1-thio-β-D-galactopyranoside (IPTG) for 18 h, and the cells were harvested by centrifugation. The cell pellets were resuspended and sonicated in 20 mM HEPES–KOH buffer (pH 7.5), containing 100 mM KCl, 5 % glycerol, 10 mM 2-mercaptoethanol, and protease inhibitor cocktail. After centrifugation at 4 °C, the supernatant was purified by passage through a Ni-Sepharose 6 Fast Flow column and a HiTrap Heparin column (GE Healthcare), followed by gel-filtration (HiLoad 16/60 Superdex 200) chromatography in 20 mM HEPES–KOH buffer (pH 7.5), containing 150 mM KCl, 5 % glycerol, and 10 mM dithiothreitol. The fractions containing eIF2A were collected, concentrated to 30 mg/mL, and stored at 4 °C.

### Crystallization and data collection

Crystals were grown at 20 °C by the sitting drop vapor diffusion method, by mixing 1 μL of protein solution with 1 μL of reservoir solution (100 mM magnesium acetate, 100 mM sodium acetate (pH 4.3), and 6 % PEG 8000). Crystals were cropped after a week. The crystals were cryoprotected with reservoir solution supplemented with 20 % propylene glycol and 5 % glycerol, and flash-cooled in liquid nitrogen. Data collection was performed at 100 K. Initial data sets were collected at the beamline station NE3A of the Photon Factory (Tsukuba, Japan), and final data sets were collected at BL41XU of SPring-8 (Harima, Japan).

### Structure determination and refinement

The data sets were initially processed with the HKL2000 program suite [11]. Among the possible space groups, *P*6_1_22 was chosen for the next step of analysis. The autoSHARP pipeline [12] was used for detecting selenium sites, phasing and density modification. The initial phases were obtained by SeMet single wavelength anomalous dispersion (SAD). Five selenium sites were found and used in phasing. The molecular model was built with ARP/wARP [13] and Buccaneer [14]. The model was modified by manual rebuilding in Coot [15] and refined by PHENIX [16]. During the modeling analysis, we found that the crystal harbors a twin in the space group *P*3_1_21 with the twin operator (-h, -k, l), and the refinement program in PHENIX estimated the twin fraction as 0.5. Therefore, the data sets were reprocessed and the modeling was resumed, using the coordinates of the half-modeled structure. TLS parameters were not used in the refinement. The surface conservation of the molecule was calculated by the ConSurf server [17] with the putative eIF2A sequences from 28 species, and the electrostatic surface potential was determined with APBS [18].

### Structure validation and deposition

The quality of the final structure was assessed using PSVS [19]. The atomic coordinates and structure factors have been deposited in the Protein Data Bank, under the accession code 3WJ9.

## Results and discussion

**Fig. 1:**
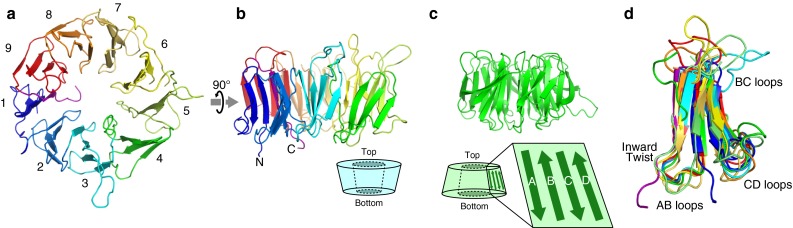
Crystal structure of the WD-repeat domain from *S. pombe* eIF2A (eIF2A-WD). **a** Top view of the structure of eIF2A-WD. Only molecule A in the asymmetric unit is shown hereafter. **b** Side view and schematic representation of the structure of eIF2A-WD. **c** An example of the canonical seven-bladed β-propeller protein (the WD-repeat region of G protein G_i_β_1_, PDB ID: 1GP2 [23] ) and schematic representation of the structure. **d** The alignment of the nine blades of eIF2A-WD

The full length *S. pombe* eIF2A (576 amino acids) was readily expressed in *E. coli*, but crystallization trials were not successful. Since the C-terminal region of eIF2A is predicted to be highly disordered, we prepared a C-terminally truncated eIF2A (residues 1–424). We successfully crystallized this truncated eIF2A, and the diffraction quality of the crystals was sufficient for a crystallographic model-building analysis. We determined the crystal structure of the C-terminally truncated eIF2A at 2.5 Å resolution (Table 1, Fig. 1a, b). The final model consists of residues 3–234, 242–248, and 256–412. The crystal structure of the truncated eIF2A revealed that it adopts a nine-bladed β-propeller fold, with the same overall topology as the regular β-propeller proteins, involving circularly arranged repeats of a four-stranded antiparallel β-sheet motif (Fig. 1a). The entire assigned region participates in the formation of the β-propeller structure, and therefore it is appropriate to define this region as the WD-repeat domain of eIF2A (eIF2A-WD). Most β-propeller proteins contain six to eight blades, and the three-dimensional structures of four- to eight-, and ten-bladed β-propellers have been determined. To our knowledge, this is the first example of a nine-bladed β-propeller protein.

**Table 1:** X-ray data collection and refinement statistics

	eIF2A-WD
Crystal parameters	
Space group	*P*3_1_21
Cell dimensions:	
a, c (Å)	93.4, 269.4
α, β, γ, (º)	90, 90, 120
Matthews coefficient (Å^3^/Da)	3.6
Solvent content (%)	66.0
Data Collection^a^	
Wavelength (Å)	0.9792
Resolution (Å)	50–2.50 (2.59–2.50)
R_sym_ (%)	8.9 (86.1)
No. of unique reflections	47,788 (4749)
No. of reflections in R_free_ set	2,392
Mean redundancy	22.1 (22.5)
Overall completeness (%)	99.7 (99.9)
Mean I/σ	49.8 (5.3)
Refinement residuals^b^	
R_free_ (%)	25.8
R_work_ (%)	23.8
Completeness (%)	99.7
Model quality	
RMSD bond lengths (Å)	0.005
RMSD bond angles (º)	0.9
Molprobity Ramachandran distribution	
Most favored (%)	95.9
Allowed (%)	4.0
Disallowed (%)	0.1
Mean main chain B-factor (Å^2^)	58.2
Mean overall B-factor (Å^2^)	60.3
Mean solvent B-factor (Å^2^)	60.4
Model contents	
Protomers in ASU	2
No. of protein atoms	6,277
No. of water molecules	61
PDB accession code	3WJ9

Entries in parentheses represent data from the limiting resolution shell. Data collection and refinement statistics were determined with *SCALEPACK* [11] and *PHENIX* [16] respectively. The abbreviations RMSD and ASU stand for root-mean-square deviation and asymmetric unit, respectively

^a^All observations with I ≥ −3 σ_I_ were included in calculating data-quality statistics

^b^Reflections with f ≥ 1.34 σ_f_ were included in calculating R-factors

There are two molecules of eIF2A-WD in the asymmetric unit, and no significant structural differences exist between them (the root-mean-square deviation is 0.98 Å for 391 Cα atoms), except for blade 6. The electron density of blade 6 is not well-defined, as compared to the other blades, due to either the crystal packing or the flexible nature of this blade (data not shown). Each of the nine blades is nearly related in the nine-fold rotational symmetry, and the oval deformation of the ring structure, as in the ten-bladed β-propeller protein Sortilin [20], is not apparent in this structure. As found in most β-propeller protein structures, one blade of eIF2A-WD is formed by the N-terminal and C-terminal residues of the β-propeller domain, probably for the stability of the propeller fold [21]. In the case of eIF2A-WD, the most interior β-sheet (the A strand) of blade 1 is formed by the C-terminal residues, and the other strands are formed by the N-terminal residues.

Conventionally, the four strands in a blade are named A to D, from the interior of each blade. The face that contains the DA and BC loops is termed the ‘top’ face, and the opposite face is termed the ‘bottom’ face (Fig. 1c). Usually, the funnel-shaped central channel of the β-propeller proteins is wider at the bottom and narrower at the top [22]. However, the central channel of eIF2A-WD has an inverted structure, with the wider opening at the top and the narrower opening at the bottom (Fig. 1b). Since this is also observed in the structure of Sortilin [20], the inversion of the wider opening direction is probably a common feature of β-propeller proteins containing a large number of blades. Furthermore, the opening at the bottom face is constricted by the inward twists of the AB loops in eIF2A-WD (Fig. 1d).

**Fig. 2:**
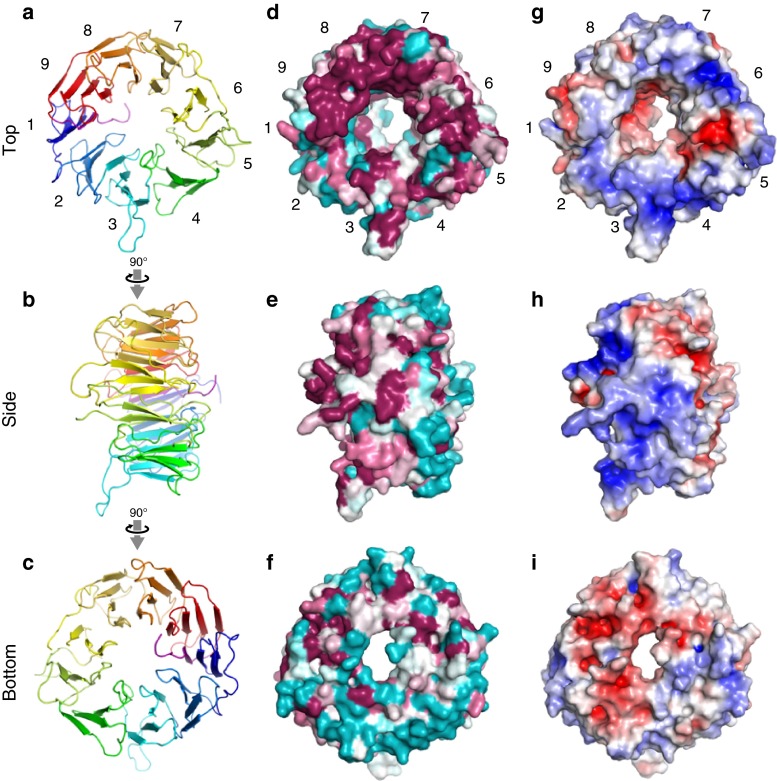
Conservation and electrostatic potential of the molecular surface of eIF2A-WD. **a**–**c** Three views of eIF2A-WD. **d**–**f** Conservation plot of the surface-exposed residues. Highly conserved and variable residues are colored *magenta* and *cyan*, respectively. **g**–**i** The electrostatic surface potential of eIF2A-WD, in which *red* and *blue colors* respectively represent negative and positive potentials of ±5 kT

Whereas the AB and CD loops at the bottom face have similar lengths and structures, the BC loops at the top face have various lengths and directions (Fig. 1d). A phylogenetic analysis revealed that the surface residues on the top face of eIF2A-WD, including these loops, are highly conserved, in contrast to the abundance of non-conserved residues on the bottom face (Figs. 2d–f). Especially, the residues at the top faces of blades 6–9 showed strong conservation. This highly conserved top face is positively charged (Fig. 2g), and the most positively-charged regions are the grooves between blades 3–4 and between blades 6–7. In addition, like the top face, the exterior face of blade 5 is highly conserved and positively charged (Fig. 2h). In many cases, the β-propeller domain plays an important role as a platform for protein–protein interactions. The existence of flexible long loops and the high surface-residue conservation at the top face of eIF2A-WD strongly suggest that this face plays such a platform-like role. Since eIF2A is implicated in interactions with nucleic acids, such as rRNA, tRNA, or IRES, the positive charges of the top face and the neighboring exterior face would be favorable for such interactions.

The roles of eIF2A in non-AUG initiated translation and protein synthesis under stress conditions where eIF2 is inactivated are poorly understood. The structure of eIF2A-WD will be a useful tool for deciphering the mechanisms of these unconventional modes of translation initiation. Furthermore, the knockdown of eIF2A in human cells reduces the infectivity of HCV, and inhibits the translation of alphaviral mRNA, which is not related to HCV. Therefore, blocking the association of eIF2A with the initiation complex could be a novel treatment for diverse types of viruses. From a medical viewpoint, this structure will also provide substantial information for drug development.

## Summary

We have determined the crystal structure of the WD-repeat domain of eIF2A from *Schizosaccharomyces pombe*. The structure adopts a novel nine-bladed β-propeller fold. In contrast to the usual β-propeller proteins, the central channel of this domain has the wider opening at the top and the narrower opening at the bottom. A phylogenetic analysis revealed that most of the conserved residues are located at the top face of this protein. This conserved top face is positively charged, and thus may play an important role in unconventional translation initiation.

## Abbreviations

ASU: Asymmetric unit
eIF: Eukaryotic translation initiation factor
GTP: Guanosine triphosphate
HCV: Hepatitis C virus
IPTG: Isopropyl-1-thio-β-D-galactopyranoside
IRES: Internal ribosome entry site
Met-tRNA_i_^Met^: Methionylated initiator tRNA
MHC: Major histocompatibility complex
PDB: Protein Data Bank
SeMet: Selenomethionine
SAD: Single wavelength anomalous dispersion
